# How Safety Climate Influences the Willingness to Stay of Nursing Staff during the COVID-19 Outbreak

**DOI:** 10.3390/healthcare9040451

**Published:** 2021-04-12

**Authors:** Ying Wang, Changyong Liang, Shuping Zhao, Yiming Ma, Yuguang Xie

**Affiliations:** The School of Management, Hefei University of Technology, Hefei 230009, China; yingwanghfut@163.com (Y.W.); cyliang@hfut.edu.cn (C.L.); mayiming923@163.com (Y.M.); 2018110762@mail.hfut.edu.cn (Y.X.)

**Keywords:** safety climate, psychological capital, nursing staff in the aged care industry, willingness to stay

## Abstract

The outbreak of COVID-19 in China at the beginning of 2020 has made the problems that the aged care agency face with large mobility and high turnover of aged nursing staff become more serious. Aiming at this problem, this paper incorporates psychological capital and social panic into the model from the perspective of the organizational safety climate and constructs a theoretical model of the mechanism of the effect on nursing staff’s willingness to stay in the context of the outbreak. Through a questionnaire survey in an aged care agency in Anhui Province, a total of 321 valid questionnaires were collected for empirical analysis. The results show that: (1) the safety climate of the organization has a significant positive impact on the transactional psychological capital and interpersonal psychological capital of nursing staff in the aged care industry and their willingness to stay; (2) transactional psychological capital and social panic have a significant positive impact on the willingness to stay of nursing staff, while interpersonal psychological capital has no significant impact on the willingness to stay; (3) the mediating role of transactional psychological capital and interpersonal psychological capital between the safety climate and the willingness to stay is established, and the moderating role of social panic between psychological capital and willingness to stay is also established. Finally, based on the research conclusions, corresponding countermeasures and suggestions are put forward to deal with the problems that occur in special periods.

## 1. Introduction

At present, China is accelerating the implementation of the “Healthy China 2030” strategy to promote the health of the whole population before 2030. The ranking of occupations with labor shortages released by the Ministry of Human Resources and Social Security in November 2019 shows that nursing staff in the aged care industry ranked 22nd, which indicates a large gap in employees in the elderly care industry. At the same time, the nature of the work and the intensity of the labor of nursing staff in the aged care industry lead to low job satisfaction and a high employee turnover rate. According to Wu’s 2017 sample survey of aged care institutions across the country, the satisfaction rate of nursing staff for their nursing care work in the aged care industry is 69.4%, and the turnover rate is 60.6% [1]. How to improve the job satisfaction of nursing staff in the aged care industry, and reduce their turnover intentions and rate, has become an important issue that needs to be resolved in the development of the country’s health care industry.

At the beginning of 2020, the outbreak of a new type of coronavirus pneumonia in China caused tens of thousands of infections and thousands of deaths. To cut off the possible spread of the new coronavirus, aged care institutions across the country implemented the requirement whole staff to stay on-site 100% of the time. This condition is a huge challenge for nursing staff in aged care agencies: a closed service environment not only increases the intensity of their daily nursing care services, but also greatly increases their psychological pressure, and will significantly impact the willingness of nursing staff in the aged care industry to stay in their jobs.

Previous studies on employee turnover intentions have focused on general research scenarios [2]; few scholars have considered the impact of changes in the social environment. Previous studies on organizational climate are more focused on high-risk industries such as coal mining, electric power, and construction [3,4,5], with a lack of research on organizational safety climates similar to that during the COVID-19 outbreak, to study employees’ willingness to stay. The aged care industry is a high-risk occupation during the outbreak of COVID-19. Therefore, the organizational safety climate during the COVID-19 outbreak is of great significance to nursing staff in the aged care industry. At the same time, the social panic caused by the COVID-19 incident also had an impact on the willingness to stay of nursing staff. Social panic refers to the unreasonable and irrational behavior and psychological reactions of the public in the face of real or imaginary threats in the face of major social emergencies. It is reflected by a group panic and has a higher “infectivity” compared with individual panic. Moreover, an individual’s positive mental state, that is, his or her psychological capital, significantly impacts his or her behavior [6,7], but few scholars have studied this in combination with the special social environment.

Social cognitive theory shows that individuals, environment, and behavior are interrelated and influence each other [8]. Human behavior is not only affected by the environment, but it will also be affected by individual psychological effects. Combined with social cognitive theory, this research focuses on two issues:

(1) In a specific social environment (during the outbreak of COVID-19), does the safety climate of aged care institutions and the perception of social panic by the nursing staff in the institutions affect their willingness to stay?
(2) Does the psychological capital of nursing staff in the aged care industry affect their willingness to stay, and what is its role in the process of the safety climate affecting the willingness to stay?

## 2. Background

### 2.1. Organizational Safety Climate and Psychological Capital

The concept of safety climate was first proposed by Israeli scholar Zohar, who pointed out that safety climate refers to the common perception of employees in an organization about the dangers of the working environment in the organization [4]. Psychological capital has been developed on the basis of positive psychology theory and positive organizational behavior theory. Ke and others conducted localized research in the Chinese context and developed a localized scale to divide psychological capital into two dimensions: transactional psychological capital and interpersonal psychological capital [9]. Transactional psychological capital includes four aspects: self-confidence and bravery, optimistic hope, willingness to forge ahead, and tenacity, which are similar to the Western definition of psychological capital. Interpersonal psychological capital includes four aspects: modesty and sincerity, tolerance and forgiveness, gratitude and dedication, and respect and courtesy.

The research of Avey et al. also showed that a good organizational climate can significantly affect psychological capital, which in turn affects individual work performance [6]. Through specific data analysis, Bergheim et al. found evidence that the organizational safety climate is directly related to individual psychological capital [10]. Therefore, the safety climate of the organization can ensure the stability and safety of the employees’ working environment, which in turn can allow employees to have more positive emotions, affecting their psychological capital [11], and thus affecting their willingness to work and their behavior. Especially in the period of the current outbreak, ensuring the individual’s perception of the organization’s safety climate might effectively improve the individual’s positive mental state. Based on the above, the following hypothesis is proposed:

**Hypothesis** **1** **(H1).** *The safety climate of aged care institutions during the COVID-19 outbreak has a significant positive impact on the psychological capital of nursing staff in the aged care industry.*

### 2.2. Safety Climate and Willingness to Stay

Willingness to stay is an important concept in human resource management. It is the initial stage of individual turnover behavior and has great impacts for individual turnover behavior [12]. Previous studies have found that the safety climate of an organization can not only effectively predict the safety behavior of an individual, but also have a positive impact on the individual’s psychology, such as by improving individual job satisfaction, and reducing job pressure and willingness to leave [13]. In the case of hidden dangers in the social environment, the individual’s willingness to leave will increase significantly due to changes in the social environment, but a good organizational safety climate will prompt individuals to have a positive mindset. As the organizational safety climate improves, the individual’s willingness to leave will decrease [14]. Through empirical research, Smith found that the safety climate has a significant positive impact on employees’ job satisfaction, while both the safety climate and job satisfaction have a significant negative impact on individuals’ willingness to leave [15]. Based on the above, the following hypothesis is proposed:

**Hypothesis** **2** **(H2).** *The safety climate of aged care institutions during the COVID-19 outbreak has a significant positive impact on the willingness of nursing staff in the aged care industry to stay.*

### 2.3. Psychological Capital and Willingness to Stay

Factors such as job satisfaction, organizational commitment, and willingness to stay have an important impact on employees’ work attitudes [16,17], and play an important role for both employees and organizations. Luthans and Jensen conducted research on hospital nurses and found that the psychological capital of nurses’ self-assessment has a significant positive impact on their willingness to stay and their emotional commitments. A positive mental state often promotes individuals to stay in the organization and take advantage of the value of long-term employment, achieving professional success for both individuals and organizations [18]. Avey et al. showed that an individual’s psychological capital has a significant positive impact on positively oriented work attitudes (such as job satisfaction, professional happiness, etc.), while negatively oriented work attitudes (such as willingness to leave, work pressure, etc.) are negatively correlated [19]. Therefore, in the context of an outbreak, it is important for individuals to maintain a positive mental state, which has a significant impact on improving employee job satisfaction and organizational commitment and reducing turnover intentions [20,21,22]. Based on the above, the following hypothesis is proposed:

**Hypothesis** **3** **(H3).** *The psychological capital of elderly care workers during the COVID-19 outbreak has a significant positive impact on their willingness to stay*.

### 2.4. The Mediating Role of Psychological Capital

Psychological capital theory shows that psychological capital plays a mediating role in the process of organizational environmental factors affecting employees’ behavioral willingness. Luthans and Avolio confirmed the mediating relationship between psychological capital in the organizational climate and individual work results [23]. At the same time, Avey et al. also verified the mediating relationship between psychological capital in the organizational climate and employee performance [24]. Based on the above, the following hypothesis is proposed:

**Hypothesis** **4** **(H4).** *The psychological capital of nursing staff in the aged care industry during the COVID-19 outbreak has a mediating effect on the organizational safety climate and the willingness to stay.*

### 2.5. The Direct and Moderating Effects of Social Panic

An individual’s panic is caused by panic psychology, which is generally a psychological response to major social risk events, such as a major natural disaster, the SARS virus outbreak, or the outbreak of the new coronary pneumonia. Panic affects individuals’ public decisions and rational judgments [25]. The study by Law et al. also found that the panic of a crowd is more likely to be generated and spread than the panic of one person, when facing major social emergencies, and thus has a higher “infectiousness” [26], which can easily cause social panic. This kind of social panic often has an important influence on an individual’s perception of a certain thing or decision of a certain behavior. During the COVID-19 outbreak, considering the closed management of aged care institutions, a greater degree of social panic perceived by the elderly care staff might lead to greater safety in staying in the aged care institutions and thus a greater willingness to stay. Therefore, based on the above content, the following hypotheses are proposed:

**Hypothesis** **5** **(H5).** *Perception of social panic among nursing staff in the aged care industry during the COVID-19 outbreak has a significant positive impact on their willingness to stay.*

**Hypothesis** **6** **(H6).** *Perception of social panic among nursing staff in the aged care industry during the COVID-19 outbreak has a moderating effect between psychological capital and willingness to stay.*

**Figure 1:**
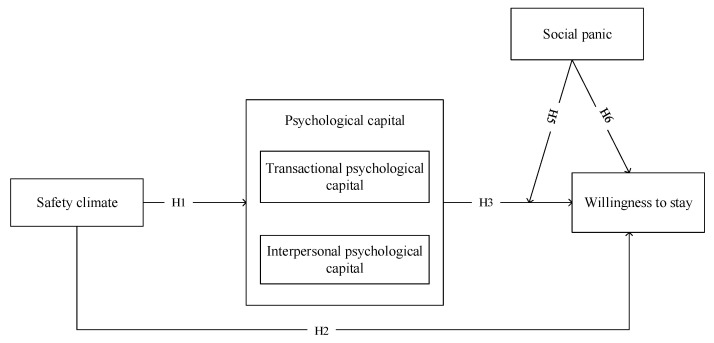
Model of the theoretical structure.

The model is shown in Figure 1.

## 3. Methods

### 3.1. Measures

In order to verify the rationality and validity of the theoretical model proposed in this article, we distributed online questionnaires to nursing staff in the aged care industry in some elderly care institutions in Anhui Province, China. The questionnaire was in the form of a Likert 7-level scale, with 1 to 7 representing the degree of approval of the respondent for the question, with 1 representing strong disapproval, and 7 representing strong approval. 

The safety climate (SC) items are based on the Hospital Safety Climate Scale for blood-borne occupational exposure compiled by Gershon et al., translated by Xu. The Chinese version of this scale has five dimensions, namely feedback and training, cleanliness and tidiness, obstacles to safe work, management support, conflict and communication, and a total of 14 measurement items. Psychological capital is based on the localized scale developed by Ke Jianglin and others for localized research in the Chinese context. It divides psychological capital into two dimensions: transactional psychological capital (TPC) and interpersonal psychological capital (IPC), with a total of 30 measurement items [9]. Social panic (SP) is based on Liu Ying’s research results and modified on this basis. There are 6 measurement items in total [27]. Finally, the willingness to stay (WTS) is modified on the basis of the Chinese version of the nurses’ willingness to stay scale developed by Tao and Wang [28], a total of three measurement items.

### 3.2. Sample Selection and Data Collection

This study was conducted on nursing staff in the aged care industry in aged care institutions in Anhui Province after the outbreak of COVID-19. First, in order to verify the validity of the questionnaire, 50 questionnaires were issued in the early stage as a pre-survey, and the questionnaire items were appropriately modified according to the results of the pre-survey. Subsequently, the revised and improved questionnaires were distributed and collected on a large scale. The subjects of the survey were nursing staff in the aged care industry in aged care institutions. After two rounds of questionnaires, a total of 350 questionnaires were distributed in this survey. After excluding invalid questionnaires, a total of 321 valid questionnaires were returned, with an effective rate of 91.71%. The specific data collection is shown in Table 1.

### 3.3. Ethics

Regarding the issue of ethical approval, we consulted the Institutional Review Board (IRB) before the data collection. The IRB carefully checked our study including questionnaire, and informed that ethical approval was unnecessary for this study. Our study is based on the perspective of management, and we did not collect any private information of participants such as personnel names, addresses, mobile phone numbers, etc. The participants signed an informed consent form and then completed the questionnaires. Additionally, the collected data are also fully confidential.

## 4. Results

### 4.1. Measurement Model

This article uses SPSS (SPSS Inc., Chicago, IL, USA) and SmartPLS (SmartPLS GmbH, Boenningstedt, Germany) software for analysis and evaluates the rationality of the measurement model and the structural model through the structural equation model of partial least squares regression.

#### 4.1.1. Descriptive Statistical Analysis

The first step is to perform a descriptive statistical analysis of the sample data. The analysis results are shown in Table 2. According to the analysis results, the mean value of each variable measurement item of the model ranges from 4.66 to 5.218, and the standard deviation ranges from 1.384 to 1.874, indicating that the data are relatively concentrated, with small fluctuations, and have good adaptability. The factor loading range of each measurement item is from 0.737 to 0.909, values that are all higher than 0.7, indicating that each variable has strong convergent validity and that there is a high correlation between the variable and the measurement item to which it belongs. Thus, the items are reasonable.

#### 4.1.2. Reliability and Validity of the Measures

First, we consider the reliability test of the questionnaire. The Cronbach’s α value reflects the reliability of each variable in the questionnaire. The higher the value, the higher the reliability of the scale. The Cronbach’s α values of all variables in this measurement model are greater than the recommended value of 0.7, and they range from 0.864 to 0.963, indicating that the reliability of the questionnaire is very good.

Next, we consider the validity test of the questionnaire. First, we test aggregate validity. Aggregate validity is a test for the consistency of multiple measurement items that are measuring the same variable. Hair and others showed that factor loading, composite reliability (CR), and average variance extraction (AVE) are the effective indicators for testing aggregate validity. The factor loading of the model is shown in Table 2. The factor loading of each variable exceeds the recommended value of 0.6. The CR value reflects the degree to which the observed variable determines the underlying structure, and also reflects the degree of internal consistency of the variable. The CR value of the model ranges from 0.901 to 0.967, which exceeds the recommended value of 0.7, indicating that the internal consistency of the model variables is good. The set observation variables can better explain the underlying model structure. The AVE value reflects the overall amount of difference in the indicators occupied by the underlying structure. The AVE in this model is in the range of 0.603 to 0.787, which exceeds the recommended value of 0.5.

Next, we test discriminant validity. Discriminant validity refers to the degree to which an observation variable does not reflect other variables. Discriminant validity can be measured by the square root of each observation variable’s AVE. In this model, the square root of each observation variable’s AVE is greater than its correlation with other observation variables’ coefficients, which shows that each observation variable has a strong discriminant coefficient and a high degree of discrimination.

The above analysis shows that the questionnaire design of this study is reasonable and has good reliability and validity. The evaluation structure of the measurement model is effective and reasonable. The measurement indicators are shown in Table 3.

### 4.2. Structural Model

**Figure 2:**
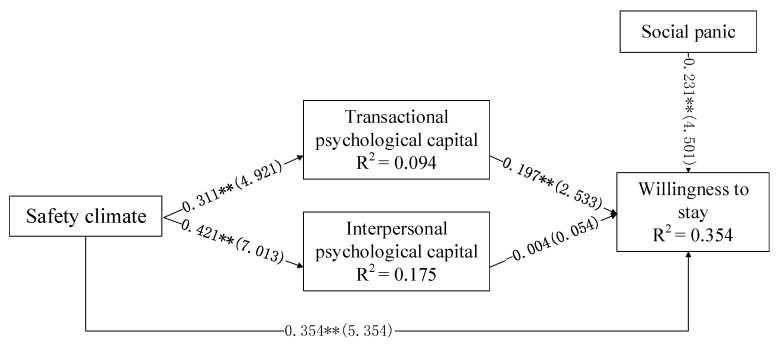
Structural model test results. Note: ** *p* < 0.01.

The structural model shows the causal relationship between the variables in the model [29]. This research uses SmartPLS software to perform structural equation analysis on the proposed theoretical model. The analysis results are shown in Figure 2.

The above model fitting shows the following: first, the safety climate of aged care institutions during the COVID-19 outbreak has a significant positive impact on the psychological capital of nursing staff in the aged care industry. The safety climate has a significant positive impact on transactional psychological capital (β = 0.311, *p* < 0.01) and interpersonal psychological capital (β = 0.421, *p* < 0.01). This shows that, in the context of the outbreak of COVID-19, the organizational safety climate perceived by nursing staff in the aged care industry can effectively enhance their inner positive psychological state. H1 is verified.

Second, psychological capital has an impact on the willingness of nursing staff in the aged care industry to retain employment. Transactional psychological capital (β = 0.197, *p* > 0.01) has a significant positive impact on the willingness of nursing staff in the aged care industry to retain employment. H3 is partial verified. Further analysis of the mediating effect of psychological capital is needed.

Finally, the safety climate (β = 0.354, *p* < 0.01) has a significant impact on the willingness of nursing staff in the aged care industry to stay at work. H2 is verified. In addition, the research results show that social panic (β = 0.231, *p* < 0.01) also has a significant positive impact on the willingness of the nursing staff to retain employment, and the co-explanatory variance of psychological capital and safety climate on the willingness to retain employment is 33.4%. H5 is verified. 

#### 4.2.1. Mediation Effect

First, we verify the mediating role of psychological capital. In order to test the mediating effect of psychological capital, this study established four models and used SmartPLS software for structural model analysis. Model 1 includes only safety climate and willingness to retain a job to analyze the impact of direct effects; Model 2 adds a transactional psychological capital variable to Model 1 to test the mediating role of transactional psychological capital; Model 3 adds an interpersonal psychological capital variable to Model 1 to test the mediating effect of interpersonal psychological capital; and Model 4 adds both transactional psychological capital and interpersonal psychological capital variables to Model 1 to test the mediating effect when both exist simultaneously.

In Model 1, the organizational safety climate (β = 0.529, *p* < 0.001) has a significant positive influence on the willingness of nursing staff in the aged care industry to stay, and the mediating effect of psychological capital can be considered [30].

In Model 2, the results show that transactional psychological capital acts as a mediator between the safety climate and the willingness of nursing staff in the aged care industry to stay. Compared to Model 1, the regression coefficient between safety climate and willingness to stay after adding the transactional psychological capital variable is reduced from 0.529 to 0.462, and the T value is reduced from 9.690 to7.526, which shows that transactional psychological capital plays a mediating role between the organizational safety climate and willingness to stay. 

In Model 3, the results show that interpersonal psychological capital acts as a mediator between the safety climate and the willingness of nursing staff in the aged care industry to stay. Compared to Model 1, the regression coefficient between safety climate and willingness to stay after adding the interpersonal psychological capital variable is reduced from 0.529 to 0.472, and the T value is reduced from 9.690 to 7.634, which shows that interpersonal psychological capital plays a mediating role between the organizational safety climate and willingness to stay. 

In Model 4, the results show that when transactional psychological capital and interpersonal psychological capital variables are added at the same time, the influence of interpersonal psychological capital on the willingness to stay is no longer significant. However, transactional psychological capital is still an important variable that affects the willingness of nursing staff in the aged care industry to stay. In summary, H4 is verified.

#### 4.2.2. Moderation Effect

**Figure 3:**
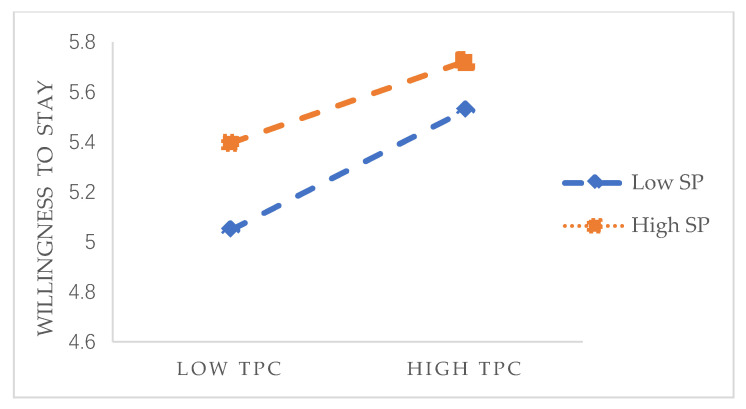
Moderating effect 1.

Then, we verify the moderating effect of social panic. SPSS (SPSS Inc., Chicago, IL, USA) analysis was used to study the moderating effect of the degree of individual perceived social panic on the relationship between transactional psychological capital and willingness to stay. The results are shown in Table 4. The results show that when the interaction term is added to the model, the interaction term is also significant (β = −0.144, *p* < 0.01). Figure 3 shows that when the individual’s perceived social panic is low, there is a strong positive correlation between the transactional psychological capital of nursing staff in the aged care industry and their willingness to stay, while the overall willingness to stay is low. When the individual’s perceived social panic is high, the positive correlation between the transactional psychological capital of the nursing staff and the willingness to stay is weaker, and the overall willingness to stay is higher. Therefore, social panic plays a negative role in the adjustment of transactional psychological capital and willingness to stay, which is in line with the original expectations. 

**Figure 4:**
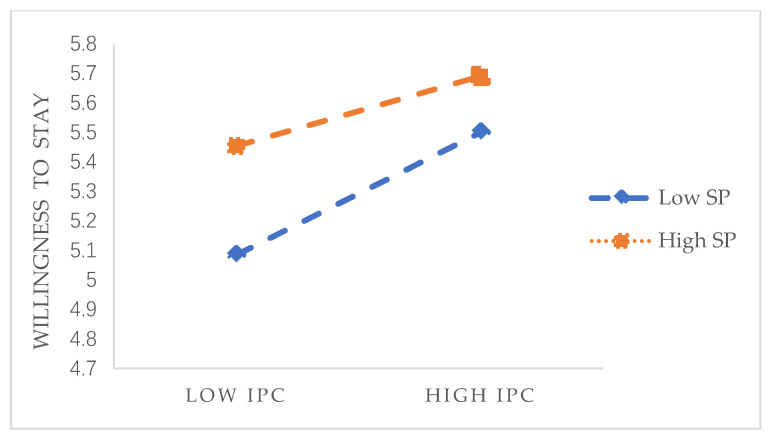
Moderating effect 2.

Similarly, Table 5 reveals the results of the moderating effect of social panic perceived by individuals on the relationship between interpersonal psychological capital and willingness to stay. Moreover, as shown in Figure 4, when social panic is low, the positive correlation between the interpersonal psychological capital of elderly care workers and the willingness to stay is stronger, and the overall willingness to stay is lower. When social panic is high, the positive correlation between the interpersonal psychological capital of the nursing staff and the willingness to stay is weaker, and the overall willingness to stay is higher. According to the results, social panic has a moderating effect on transactional psychological capital, interpersonal psychological capital and willingness to stay. The specific moderating effect results are shown in Figure 4. Additionally, H6 is verified.

According to the above analysis, we summarize all the hypothetical results, and the final structure parameter estimates results are shown in Table 6.

## 5. Discussion 

### 5.1. Main Findings

First, the research results show that the organizational safety climate can effectively improve the psychological capital of the nursing staff, including transactional psychological capital and interpersonal psychological capital. This result aligns with results that show that the safety climate of the organization can ensure the stability and safety of the working environment of the nursing staff in the aged care industry, reduce the impact of COVID-19 on employees, and allow employees to generate more positive emotions, thus affecting their psychological capital [11], which in turn impacts the individual’s work willingness and behavior. Moreover, the stronger the individual’s perception of the safety climate of the elderly care institution, the stronger their willingness to stay in the organization, which is consistent with the research results of McCaughey et al. and Wang and Yen [13,14].

Second, when considering the results of the overall model analysis, transactional psychological capital has a significant positive impact, while the influence of interpersonal psychological capital on its willingness to stay behind disappears. This result differs from the results of Ke and others [9]. The impact disappears because transactional psychological capital focuses on the positive mindset of individuals for the content of their work, while interpersonal psychological capital focuses more on the individual’s interpersonal communication at work [9]. The results also show that in the special period of the COVID-19 outbreak, employees who can better handle interpersonal relationships do not show an increase in their willingness to stay, whereas employees with rich transactional psychological capital will consider the development of both themselves and the organization and be more willing to stay in the organization and continue to play their intrinsic role.

Third, after adding transactional psychological capital and interpersonal psychological capital, the impact of the organizational safety climate on the willingness of nursing staff in the aged care industry to stay at work is reduced, indicating that the individual’s environmental perception during the outbreak of COVID-19 will directly affect their psychological state, which in turn affects their willingness to act [8]. The mediating effects of transactional psychological capital and interpersonal psychological capital have been verified. This study also confirms the research of Luthans et al. and Avey et al. [11,24].

Fourth, the perception of social panic among nursing staff in the aged care industry has a significant positive impact on their willingness to stay. This may be due to the fact that, during the COVID-19 outbreak, the safety of the relatively closed aged care institutions is higher, and elderly care personnel are therefore more willing to stay in their posts, rather than taking risks to go into society and find other jobs. In addition, the study of this paper finds that the moderating effect of social panic is valid, and it negatively moderates the relationship between psychological capital and the willingness to stay. This means that when the degree of social panic is high, the effect of psychological capital on the willingness to stay will be weakened.

### 5.2. Limitations 

This article has theoretical value and practical significance, but it also has some shortcomings: first, this article studied nursing staff in the aged care industry of an elderly care company in Anhui Province, China. It has certain geographical limitations and research object limitations. Due to the different severity of COVID-19 in different regions, the mechanism of influence on the willingness of nursing staff in the aged care industry to stay is not necessarily the same. The effective sample size collected in this article is 271, which is small. Second, this study considers the organizational safety climate; however, this is only one of the many factors that affect the willingness of nursing staff in the aged care industry to stay. It is verified in a specific context, and further improvements are needed in the future.

## 6. Conclusions

Based on social cognitive theory, positive psychology, and positive organizational behavior, this article explores the mechanism of influence on the psychological capital and willingness to stay employed of nursing staff in the aged care industry from the perspective of the organizational safety climate in the context of the outbreak of COVID-19. Nursing management during this period is of great significance. During the COVID-19 outbreak, nursing staff in aged care institutions faced greater pressure both physically and mentally, resulting in a significant increase in their turnover rate. For aged care institutions, improving the safety climate within the organization has a significant positive impact on the psychological capital of nursing staff and their willingness to stay. 

## Figures and Tables

**Table 1:** Sample Statistics of Nursing Staff in the Aged Care Industry (N = 321).

Item	Characteristic	Number	Percent
Sex	Male	78	24.30%
	Female	243	75.70%
Age	≤25 years	64	19.93%
	26–35 years	74	23.06%
	36–45 years	130	40.5%
	≥46 years	53	16.51%
Education background	High school/secondary school and below	170	52.96%
	College	79	24.61%
	Undergraduate and above	67	20.87%
	Master’s degree	5	1.56%
Working years	0–3 months	56	17.45%
	3–6 months	16	4.98%
	6 months to 1 year	58	18.07%
	1–2 years	79	24.61%
	>2 years	112	34.89%
Monthly income (USD)	≤360.75	60	18.69%
	360.76–505.05	52	16.20%
	505.06–649.35	128	39.88%
	649.36–793.65	59	18.38%
	≥793.66	22	6.85%

**Table 2:** Descriptive Statistical Analysis.

Variable	Item	Mean Value	Standard Deviation	Factor Loading
Safety climate	SC1	5.009	1.582	0.774
	SC2	4.769	1.384	0.756
	SC3	4.844	1.51	0.84
	SC4	4.984	1.578	0.862
	SC5	4.947	1.496	0.856
	SC6	4.863	1.551	0.816
	SC7	4.956	1.465	0.821
	SC8	4.966	1.537	0.82
	SC9	5.019	1.575	0.819
	SC10	5.075	1.557	0.786
	SC11	5.218	1.564	0.796
	SC12	5.112	1.585	0.789
	SC13	5.022	1.574	0.808
	SC14	5.019	1.537	0.799
Transactional Psychological Capital	TPC1	5.047	1.656	0.833
	TPC2	4.857	1.642	0.787
	TPC3	4.95	1.592	0.795
	TPC4	5.019	1.549	0.801
	TPC5	5.056	1.609	0.832
	TPC6	5.016	1.572	0.799
	TPC7	5.09	1.579	0.809
	TPC8	5.137	1.583	0.801
	TPC9	5.053	1.571	0.801
	TPC10	4.991	1.586	0.766
	TPC11	4.956	1.507	0.818
	TPC12	4.972	1.609	0.818
	TPC13	5.04	1.553	0.809
Interpersonal Psychological Capital	IPC1	4.991	1.588	0.79
	IPC2	5.072	1.503	0.806
	IPC3	5.037	1.592	0.802
	IPC4	5.012	1.573	0.802
	IPC5	4.96	1.579	0.792
	IPC6	4.804	1.575	0.761
	IPC7	4.953	1.608	0.763
	IPC8	5.053	1.573	0.771
	IPC9	5.034	1.694	0.827
	IPC10	5.006	1.614	0.813
	IPC11	4.988	1.575	0.788
	IPC12	4.91	1.641	0.777
	IPC13	4.963	1.55	0.776
	IPC14	4.95	1.599	0.805
	IPC15	4.872	1.574	0.797
	IPC16	4.854	1.652	0.829
	IPC17	4.931	1.608	0.786
Social Panic	SP1	4.754	1.832	0.738
	SP2	4.745	1.844	0.78
	SP3	4.701	1.874	0.751
	SP4	4.66	1.793	0.737
	SP5	4.984	1.58	0.834
	SP6	5.028	1.585	0.812
Willingness to Stay	WTS1	4.738	1.502	0.842
	WTS2	4.763	1.489	0.908
	WTS3	4.879	1.406	0.909

**Table 3:** Reliability and Validity Tests.

Item	Alpha	CR	AVE	TPC	IPC	SC	WTS	SP
TPC	0.955	0.96	0.649	0.805				
IPC	0.963	0.967	0.63	0.671	0.793			
SC	0.96	0.964	0.657	0.311	0.421	0.811		
WTS	0.864	0.917	0.787	0.361	0.333	0.529	0.887	
SP	0.885	0.901	0.603	0.243	0.24	0.498	0.454	0.776

**Table 4:** Moderating Effect Test 1.

Variable	Willingness to Stay		Willingness to Stay	
	Standardized Coefficient	T Value	Standardized Coefficient	T Value
Sex	–0.016	–0.287	–0.013	–0.235
Age	–0.068	–1.525	–0.069	–1.41
Education background	0.034	0.510	0.047	0.911
Working years	–0.089	–1.756	–0.086	–1.628
Transactional psychological capital	0.341 **	6.507	0.314	5.923
Social panic	0.109 *	2.080	0.092	1.750
Transactional psychological capital and social panic			−0.144	−2.707
R^2^	0.132		0. 149	
F value	25.346		19.677	

Note: * *p* < 0.05; ** *p* < 0.01.

**Table 5:** Moderating Effect Test 2.

Variable	Willingness to Stay		Willingness to Stay	
	Standardized Coefficient	T Value	Standardized Coefficient	T Value
Sex	–0.02	–0.367	–0.016	–0.238
Age	–0.089	–1.735	–0.086	–1.688
Education background	0.035	0.742	0.068	1.281
Working years	–0.091	–1.865	–0.09	–1.812
Interpersonal psychological capital	0.315 **	5.963	0.285 **	5.439
Social panic	0.123 *	2.328	0.107 *	2.048
Interpersonal psychological capital and social panic	-	-	−0.189	−3.597
R^2^	0.115		0.147	
F value	21.875		19.442	

Note: * *p* < 0.05; ** *p* < 0.01.

**Table 6:** Structure Parameter Estimates.

Hypothesized Path	Standardized Path Coefficient	T Value	*p* Value	Result
H1: Safety climate → Psychological capital	0.311 **/0.421 **	4.921/7.013	0	Supported
H2: Safety climate → Willingness to stay	0.354 *	5.354	0	Supported
H3: Psychological capital → Willingness to stay	0.197 **/−0.004	2.533/0.054	0.011/0.957	Supported
H4: The mediating role of psychological capital	-	-	-	Supported
H5: Social panic → Willingness to stay	0.231 **	4.501	0	Supported
H6: Psychological capital → Willingness to stay	-	-	-	Supported

Note: * *p* < 0.05, ** *p* < 0.01.

## Data Availability

The data presented in this study are available on reasonable request from the author.

## References

[1] Wu Z., Zhang F., Tonggui T. (2017). Survey on the status quo of nursing staff in nursing homes. Soc. Welf..

[2] Cowin L.S., Johnson M., Craven R.G., Marsh H.W. (2008). Causal modeling of self-concept, job satisfaction, and retention of nurses. Int. J. Nurs. Stud..

[3] Cox S., Cheyne A. (2000). Assessing safety culture in offshore environments. Saf. Sci..

[4] Zohar D. (1980). Safety climate in industrial organizations: Theoretical and applied implications. J. Appl. Psychol..

[5] Zohar D., Luria G. (2005). A Multilevel Model of Safety Climate: Cross-Level Relationships Between Organization and Group-Level Climates. J. Appl. Psychol..

[6] Avey J.B., Wernsing T.S., Luthans F. (2008). Can Positive Employees Help Positive Organizational Change? Impact of Psychological Capital and Emotions on Relevant Attitudes and Behaviors. J. Appl. Behav. Sci..

[7] Avolio B.J., Zhu W., Koh W., Bhatia P. (2004). Transformational leadership and organizational commitment: Mediating role of psychological empowerment and moder-ating role of structural distance. J. Organ. Behav. Int. J. Ind. Occup. Organ. Psychol. Behav..

[8] Grusec J.E. (2020). Social Learning Theory and Developmental Psychology: The Legacies of Robert R. Sears and Albert Bandura. Int. J. Dev. Sci..

[9] Jianglin K., Dan W., Jianmin S. (2015). The influence of psychological capital on work engagement, subjective well-being and silent be-havior: A comparison of interaction effects and effects. Psychol. Behav. Res..

[10] Bergheim K., Eid J., Hystad S.W., Nielsen M.B., Mearns K., Larsson G., Luthans B. (2013). The Role of Psychological Capital in Perception of Safety Climate among Air Traffic Controllers. J. Leadersh. Organ. Stud..

[11] Luthans F., Avolio B.J., Avey J.B., Norman S.M. (2007). Positive psychological capital: Measurement and relationship with performance and satisfaction. Pers. Psychol..

[12] Laschinger H.K.S., Grau A.L. (2012). The influence of personal dispositional factors and organizational resources on workplace violence, burnout, and health outcomes in new graduate nurses: A cross-sectional study. Int. J. Nurs. Stud..

[13] McCaughey D., DelliFraine J.L., McGhan G., Bruning N.S. (2013). The negative effects of workplace injury and illness on workplace safety climate perceptions and health care worker outcomes. Saf. Sci..

[14] Wang C.-H., Yen C.-D. (2015). Leadership and turnover intentions of Taiwan TV reporters: The moderating role of safety climate. Asian J. Commun..

[15] Smith T.D. (2018). An assessment of safety climate, job satisfaction and turnover intention relationships using a national sample of workers from the USA. Int. J. Occup. Saf. Ergon..

[16] Alharbi A.A., Dahinten V.S., Macphee M. (2020). The relationships between nurses’ work environments and emotional exhaustion, job satisfaction, and intent to leave among nurses in Saudi Arabia. J. Adv. Nurs..

[17] Chen I.-H., Brown R., Bowers B.J., Chang W.-Y. (2015). Work-to-family conflict as a mediator of the relationship between job satisfaction and turnover intention. J. Adv. Nurs..

[18] Luthans K.W., Jensen S.M. (2005). The linkage between psychological capital and commitment to organizational mission: A study of nurses. JONA J. Nurs. Adm..

[19] Avey J.B., Reichard R.J., Luthans F., Mhatre K.H. (2011). Meta-analysis of the impact of positive psychological capital on employee attitudes, behaviors, and performance. Hum. Resour. Dev. Q..

[20] Larson M., Luthans F. (2006). Potential Added Value of Psychological Capital in Predicting Work Attitudes. J. Leadersh. Organ. Stud..

[21] Abbas M., Raja U., Darr W., Bouckenooghe D. (2012). Combined Effects of Perceived Politics and Psychological Capital on Job Satisfaction, Turnover Intentions, and Performance. J. Manag..

[22] Youssef C.M., Luthans F. (2007). Positive organizational behavior in the workplace: The impact of hope, optimism, and resilience. J. Manag..

[23] Luthans F., Avolio B.J. (2009). The “point” of positive organizational behavior. J. Organ. Behav. Int. J. Ind. Occup. Organ. Psychol. Behav..

[24] Avey J.B., Luthans F., Jensen S.M. (2009). Psychological capital: A positive resource for combating employee stress and turnover. Hum. Resour. Manag..

[25] Jiangang Q. (2010). Risk panic of extreme events and its implications for administrative legal system. Chin. Law.

[26] Law K., Dauber K., Pan X. (2016). Computational Modeling of Nonadaptive Crowd Behaviors and Ergess Analysis: 2004–2005 CIFE Seed Project Report.

[27] Ying L. (2019). Research on the Influence of Group Panic on the Unsafe Evacuation Behavior of Subway Passengers.

[28] Hong T., Lin W. (2010). Revision of the Chinese version of the questionnaire on the willingness to stay for nurses. J. Second Mil. Med. Univ..

[29] Sang S., Lee J.-D., Lee J. (2010). E-government adoption in Cambodia: A partial least squares approach. Transform. Gov. People Process Policy.

[30] Zhang N., Zhou Z.-M., Su C.-T., Zhou N. (2013). How Do Different Types of Community Commitment Influence Brand Commitment? The Mediation of Brand Attachment. Cyberpsychology Behav. Soc. Netw..

